# Which role for chest x-ray score in predicting the outcome in COVID-19 pneumonia?

**DOI:** 10.1007/s00330-020-07504-2

**Published:** 2020-12-02

**Authors:** Roberto Maroldi, Paolo Rondi, Giorgio Maria Agazzi, Marco Ravanelli, Andrea Borghesi, Davide Farina

**Affiliations:** Department of Medical and Surgical Specialties, Radiological Sciences and Public Health, University of Brescia, Piazzale Spedali Civili 1, 25123 Brescia, Italy

**Keywords:** X-rays, Predictive model, Prognosis, COVID-19, Severe acute respiratory syndrome coronavirus 2

## Abstract

**Objective:**

We aim to demonstrate that a chest X-ray (CXR) scoring system for COVID-19 patients correlates with patient outcome and has a prognostic value.

**Methods:**

This retrospective study included CXRs of COVID-19 patients that reported the Brixia score, a semi-quantitative scoring system rating lung involvement from 0 to 18. The highest (H) and lowest (L) values were registered along with scores on admission (A) and end of hospitalization (E). The Brixia score was correlated with the outcome (death or discharge).

**Results:**

A total of 953 patients met inclusion criteria. In total, 677/953 were discharged and 276/953 died during hospitalization. A total of 524/953 had one CXR and 429/953 had more than one CXR. H-score was significantly higher in deceased (median, 12; IQR 9–14) compared to that in discharged patients (median, 8; IQR 5–11) (*p* < 0.0001). In 429/953 patients with multiple CXR, A-score, L-score, and E-score were higher in deceased than in discharged patients (A-score 9 vs 8; *p* = 0.039; L-score 7 vs 5; *p* < 0.0003; E-score 12 vs 7; *p* < 0.0001). In the entire cohort, logistic regression showed a significant predictive value for age (*p* < 0.0001, OR 1.13), H-score (*p* < 0.0001, OR 1.25), and gender (*p* = 0.01, male OR 1.67). AUC was 0.863. In patients with ≥ 2 CXR, A-, L-, and E-scores correlated significantly with the outcome. Cox proportional hazards regression indicated age (*p* < 0.0001, HR 4.17), H-score (< 9, HR 0.36, *p* = 0.0012), and worsening of H-score vs A score > 3 (HR 1.57, *p* = 0.0227) as associated with worse outcome.

**Conclusions:**

The Brixia score correlates strongly with disease severity and outcome; it may support the clinical decision-making, particularly in patients with moderate-to-severe signs and symptoms. The Brixia score should be incorporated in a prognostic model, which would be desirable, particularly in resource-constraint scenarios.

**Key Points:**

*• To demonstrate the importance of the Brixia score in assessing and monitoring COVID-19 lung involvement.*

*• The Brixia score strongly correlates with patient outcome and can be easily implemented in the routine reporting of CXR.*

## Introduction

In December 2019 in Wuhan (province of Hubei, China), several cases of pneumonia caused by an unknown pathogen were registered [1]. A novel virus, named severe acute respiratory syndrome coronavirus 2 (SARS-CoV-2), was isolated in samples obtained from human respiratory tract and identified as the cause of the disease, currently termed coronavirus disease 2019 (COVID-19) [2].

In the first 2 months of 2020, the virus spread across the globe involving an increasing number of nations. On March 11, the COVID-19 outbreak was declared a pandemic by the WHO when our region, Lombardy, in northern Italy, was already deeply involved in a healthcare emergency [3].

At that time, the emergency department (ED) was over flooded with patients afflicted by the symptoms of COVID-19; thus, providing an adequate response was a life-saving urgency. A camp-site extension of the ED was installed in the hospital front yard; several hospital wards were reorganized and exclusively dedicated to COVID-19 patients; intensive care units (ICU) nearly quadruplicated their capacity. Nonetheless, at the climax of the emergency, several patients had to be transferred to other hospitals in Italy and in the EU, due to shortage of ICU beds [4].

Radiologists had to face the increasing demand of chest imaging: at the beginning of the outbreak, on average 100 chest X-rays (CXRs) were performed daily, most at bedside; at the peak, the number of CXR grew up to 200 [5].

Although chest CT entails a higher sensitivity and specificity in the assessment of COVID-19-related lung anomalies [6–8], we decided to use CXR as a workhorse because the acquisition with portable equipment offered faster and logistically easier alternative [5]. In addition, this technique was better suited for serial monitoring in patients in poor clinical conditions. The *Brixia* score [9] added to the descriptive report semi-quantitative information on the severity of the lung disease and was adopted for the triage in ED and for grading lung lesions during the hospitalization [10].

The purpose of this study is to assess the clinical value of the *Brixia* score in assessing the severity of lung lesions and to explore its potential application in predictive models.

## Material and methods

**Fig. 1:**
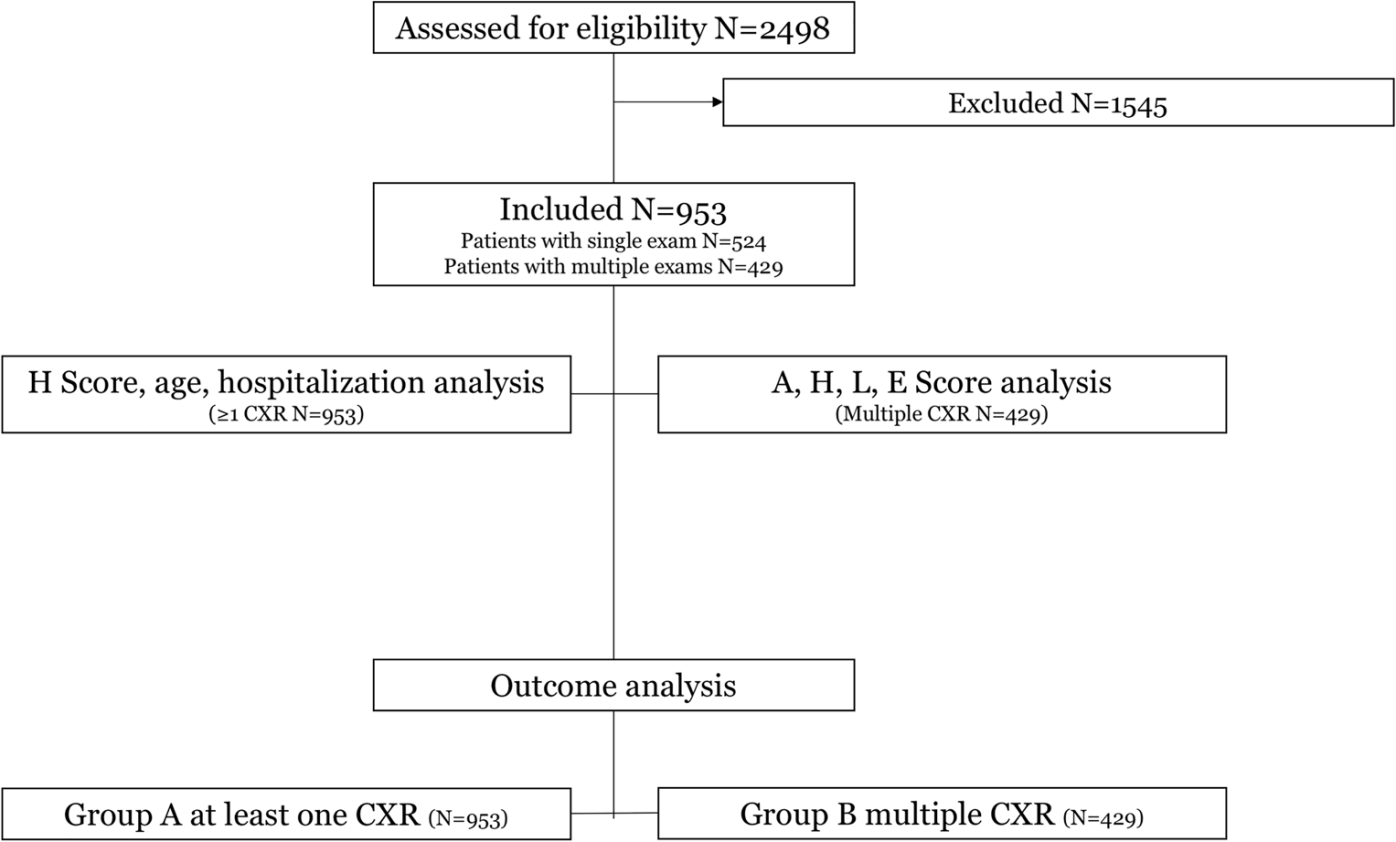
Patient selection flowchart

**Fig. 2:**
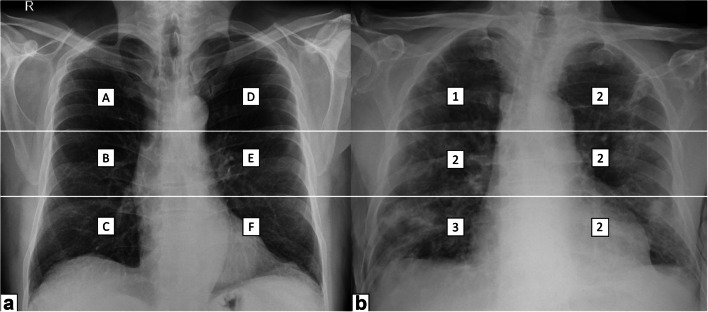
Brixia score. **a** The lung is divided into 6 zones (A–F). For each zone, a score from 0 to 3 is assigned. **b** An example of application of the scoring system in a COVID-19 patient. Brixia score = 13 (123222)

This retrospective study was conducted in accordance with the ethical standards of the Declaration of Helsinki and was approved by our local institutional review board (IRB NP 4121). Our local IRB waived the need for informed consent due to the retrospective nature of the study, which analyzes already acquired clinical and radiological data. We searched our institutional electronic health record for eligible patients, and our study population was obtained from a cohort of 2498 patients admitted at the hospital because of signs and symptoms suggestive for COVID-19 pneumonia (inclusion date between March 4, 2020, and April 11, 2020) (Fig. 1). Inclusion criteria were as follows: available CXR reporting the severity index expressed by the *Brixia* score, positivity for SARS-CoV-2 infection (confirmed by RT-PCR), and outcome classifiable by death during hospitalization or discharge to home care or to rehabilitation. Exclusion criteria were as follows: age < 18. The *Brixia* score divides each lung into three regions, from apex to base (right: ABC; left: DEF), and classifies parenchymal findings as normal (0), interstitial changes (1), interstitial and alveolar changes with interstitial predominance (2), and interstitial and alveolar changes with alveolar predominance (3). Consequently, the *Brixia* score generates an overall severity score ranging from 0 to 18; in addition, a string of 6 digits was added to each report to detail the value assigned to each region (Fig. 2).

The following Brixia scores were retrieved: the highest score (H-score), corresponding to the sole score for patients who had a single chest x-ray and to the highest score for those with multiple CXR examinations; in the latter group, we also recorded the lowest score (L-score), the score assigned at admission (A-score), and the last score obtained before the hospitalization ended (E-score) or the patient died. The following clinical characteristics were retrieved for each patient: age, sex.

Data are presented as the median and the interquartile range (IQR) because the age of the patients and the *Brixia* score were not normally distributed. The Kruskal-Wallis test was used to determine whether a significant difference for the median CXR score was present among the two outcomes. A post hoc analysis was done using the Conover test. The Mann-Whitney test was used to verify if the ranking of CXR score was different between deceased and discharged patients. The chi-square test was performed in order to assess differences in categorical variable distribution. We compared radiological and clinical characteristics among the different outcomes by means of multivariable logistic regression models, and Cox proportional hazards model. Relevant parameters were selected using a stepwise approach. We plotted a ROC curve of the final model and calculated the AUC as a measure of diagnostic accuracy. We compared differences in diagnostic accuracy between ROC curves using the DeLong test. Differences in mean time to event were compared by means of the log-rank test. Statistical analyses were performed using commercial software (MedCalc Statistical Software version 19.2.1). *P* values of < 0.05 were considered statistically significant.

## Results

A total number of 953 patients were included in the study. The patients had either one CXR (524/953, 55%) or more than one CXR (429/953, 45%) (Fig. 1 shows the process of patient selection). The latter group had a median of 3 CXRs. The age of the 1095 selected patients ranged from 20 to 98 years (median, 69; IQR, 58–78); male patients accounted for 66.1%. The majority of patients 895/953 (93.9%) were treated in isolated medical units, temporarily dedicated to COVID-19.

Of the 953 patients, 677 (71%) were discharged (605/677 to home care, 72/677 to rehabilitation, 89% and 11% respectively), and 276 (29%) died during the hospitalization.

The hospital stay ranged from 1 day to 40 (median, 9 days; IQR, 6–14). The hospitalization was longer in discharged patients (median, 11 days, IQR, 6–15 vs 7, IQR 4–12.5; *p* < 0.0001) compared to that in deceased patients.

The median age of deceased patients (median, 78 years; IQR, 73–82) was significantly higher than the age of discharged patients (median, 63 years; IQR, 54–73) (*p* < 0.0001). The post hoc analysis showed statistically significant differences (*p* < 0.05) in median age between all the two groups. Patients sent to home care were younger than those sent to rehabilitation (62 years, IQR 53–71; vs 76.5 years, IQR 65–83; *p* < 0.0001) (Tables 1 and 2).

**Table 1:** Outcome in 953 patients: age and H-score. Data are presented as medians (interquartile range)

Independent variables	Decease in hospital (276 patients)	Discharge (677 patients)	*p* value
Age	78 (73–82)	63 (54–73)	*p* < 0.0001
H-score	12 (9–14)	8 (5–11)	*p* < 0.0001

**Table 2:** Outcome in 677 patients discharged. Data are presented as medians (interquartile range) (*n.s.* not significant)

Independent variables	To rehabilitation (72 patients)	To home care (605 patients)	*p* value
Age	76.5 (65–83)	62 (53–71)	*p* < 0.0001
H-score	9 (6–12)	8 (4–11)	*p* n.s.

In the 953 patients, the H-score was significantly higher in deceased patients (median, 12; IQR 9–14) compared to that in discharged patients (median, 8; IQR 5–11) (*p* < 0.0001). The post hoc analysis confirmed statistically significant differences among the two groups (*p* < 0.05).

In the 429 patients with multiple CXR examinations, the A-score, L-score, and E-score were all higher in the 109 deceased patients than in the 320 discharged patients (A-score 9 vs 8, *p* = 0.039; L-score 7 vs 5, *p* < 0.0003; E-score 12 vs 7, *p* < 0.0001) (Table 3).

**Table 3:** Age and Brixia scores in 498 patients who underwent multiple chest x-ray examinations. Data are presented as medians (interquartile range). ^a^L-score is significantly higher in deceased patients than in discharged patients. ^b^A-score is significantly higher in deceased patients than in discharged patients. ^c^E-score in deceased patients is significantly greater than that in discharged patients

Independent variables	Decease in hospital (109 patients)	Discharge (320 patients)	*p* value
Age	76 (71–81)	62 (54–72)	*p* < 0.000001
H-score	14 (11.8–16)	10 (7–13)	*p* < 0.000001
L-score	7 (4–9.25)^a^	5 (2–8)^a^	*p* = 0.003^a^
A-score	9 (6–12)^b^	8 (4–11)^b^	*p* = 0.0398^b^
E-score	12 (9.8–15)^c^	7 (4–10)	*p* < 0.00001

A logistic regression was performed in the entire cohort of 953 patients (276 deceased in the hospital plus 677 discharged) who had at least one CXR scored (group A). In this model, a significant prediction of the outcome was observed for age (*p* < 0.0001, OR 1.13), H-score (*p* < 0.0001, OR 1.25), and gender (*p* = 0.01, male OR 1.67). This model achieved an AUC of 0.863 (95% CI 0.84–0.89). Multivariable logistic regressions performed in the subgroup of 429 patients (group B) showed that A-, L-, and E-scores (singularly combined with age and gender) correlated all significantly with the outcome. In the three models, the A-score (admission CXR) was less predictive than the L-score, the lowest value obtained during hospitalization (AUC 0.814 vs 0.823, with 95% CI of 0.77–0.85 and 0.78–0.86, respectively). The model using the E-score obtained the largest value (AUC 0.890, 95% CI 0.86–0.92). The AUC of L-score and A-score did not show a statistically significant difference (*p* > 0.05), while AUC obtained with the E-score showed statistically significant differences compared to those with the L-score (*p* = 0.0001) and A-score (*p* = 0.0001). In order to explore the weight of CXR worsening after admission, an increase of the H-score > 3 points (compared to A-score) permitted to achieve an AUC of 0.893.

**Fig. 3:**
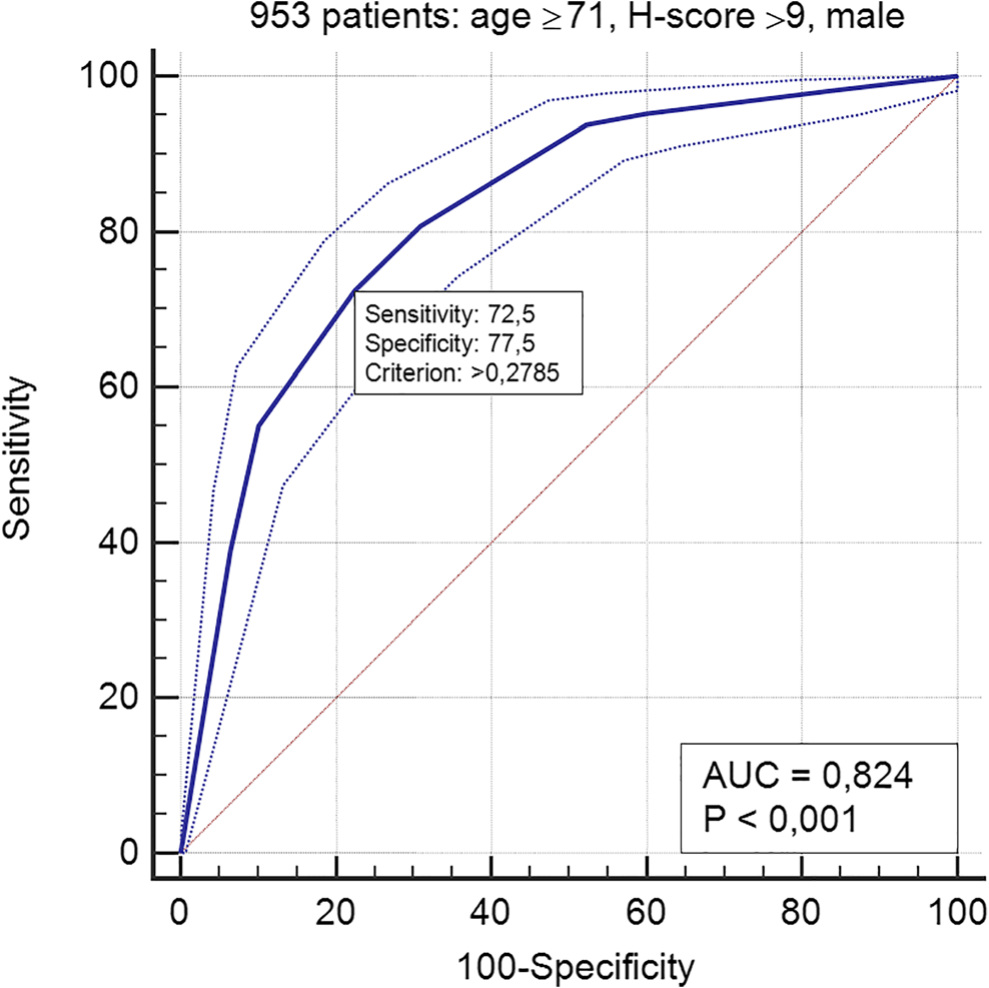
ROC curve in 953 patients: model based on age, H-score, and gender

**Fig. 4:**
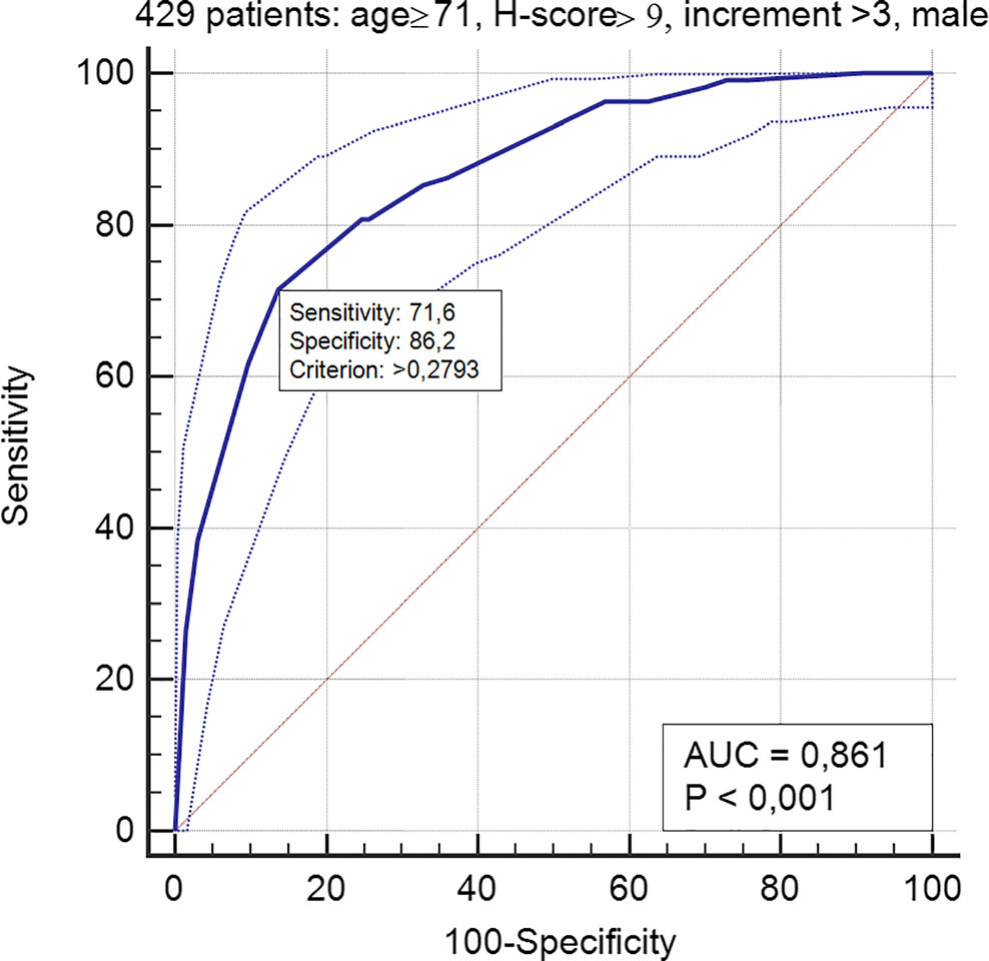
ROC curve in 429 patients: model based on age, H-score, and gender

**Fig. 5:**
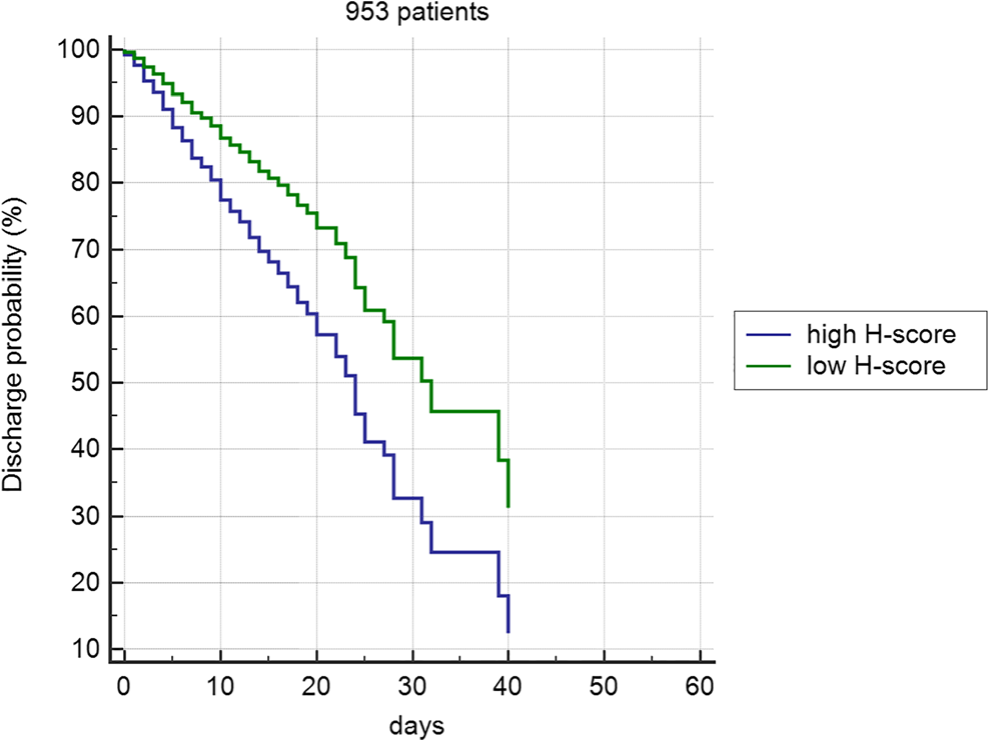
Cox proportional hazards regression in 953 patients with at least one chest x-ray. Brixia score high > 9

**Fig. 6:**
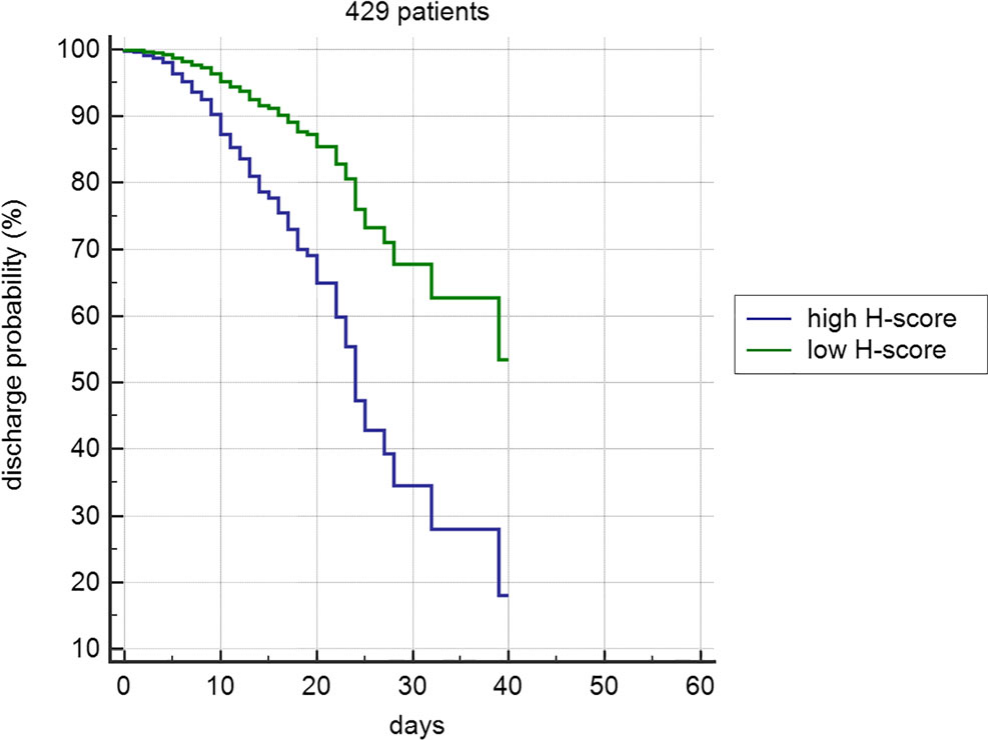
Cox proportional hazards regression in 429 patients with multiple chest x-rays. Brixia score high > 9

In group A, the ROC curve based on age ≥ 71 years, H-score > 9 (both values corresponding to the lowest IQR in deceased patients), and male gender achieved an AUC of 0.824 (Fig. 3). In group B, the ROC curve including age ≥ 71, H-score > 9, male gender, and an increase of the H-score vs A-score > 3 obtained an AUC of 0.861 (Fig. 4). The Cox proportional hazards regression indicated age, H-score threshold (in groups A and B), and worsening of H-score vs A-score > 3 (in group B) as significantly associated with the probability of the worse outcome. In group A, age HR was 5.27 (95% CI 3.9–7.1, *p* < 0.0001) and H-score threshold was 0.55 of low vs high score (95% CI 0.43–0.72, *p* < 0.0001) (Fig. 5). In group B, age HR was 4.17 (95% CI 2.7–6.5, *p* < 0.0001), H-score threshold was 0.36 of low vs high score (95% CI 0.19–0.67, *p* = 0.0012) (Fig. 6), and worsening of H-score vs A-score threshold was 1.57 when > 3 points (95% CI 1.06–2.31, *p* = 0.0227).

## Discussion

The mortality rate in the present series (29%) is significantly higher than the reported mortality rate of COVID-19. This certainly reflects the dramatic impact on the healthcare system produced by the outbreak of the disease in the epicenter of the epidemic. In fact, we analyzed a cohort of hospitalized patient in a peculiar phase, when the availability of beds (in COVID-19 units and intensive care units) modulated the admission criteria. As a consequence, the population under investigation is a selection of most severely ill patients, i.e., all affected by pneumonia and respiratory distress of variable intensity. In addition, many of the younger patients accessed the hospital after failure of home treatment (under the supervision of general practitioners), when substantial deterioration of the clinical conditions occurred.

In a scenario like the one described above, the role of imaging extends beyond the confirmation of the diagnosis, to include monitoring of the disease and, possibly, prediction of its outcome.

The *Brixia* score was conceived with the aim to provide synthetic and unequivocal information about the spatial distribution and the overall severity of the disease. The string of the six values composing the score helps the radiologist in the comparison of serial CXR, facilitating the interpretation of the report of the previous reader and providing consistent criteria of assessment. On the other hands, the summed score offers to the referring physician an immediate picture of the evolution of the disease, clearing any possible misinterpretation of a descriptive report. Though the score was rated by several radiologists in the hospital, a high interobserver agreement (0.82) was demonstrated in a cohort of 100 patients [9].

The results of this study further confirm the effectiveness of the *Brixia* score [9]. In 953 patients, the highest value reached by the score was significantly higher in deceased patients as compared to that in discharged ones. The median value of each of four different reference values of the score (representing time points or peaks in the clinical course) was significantly different (and progressively higher) in discharged and deceased patients. Wong et al recently described a scoring system for CXR findings in COVID-19 patients [11]. That score applied a simplified approach based on the percentage of lung parenchyma affected in each lung, resulting in score values ranging from 0 to 8. In our opinion, the *Brixia* score, though maintaining a reasonably easy and schematic approach, provides a more sophisticated panel of information, including the type of lung damage, its zonal distribution, and a larger range of total values, which may better grade the severity of lung involvement. According to the Multinational Consensus Statement of the Fleischner Society on the role of imaging in the COVID-19 pandemic, in patients with moderate-to-severe features of the disease, imaging is an essential part of the clinical decision-making about the intensity of care and support, even more so in a resource-constrained environment. We believe that the more refined information provided by the *Brixia* score may efficiently serve this purpose.

Resource constraints add a further complication to the difficult management of the peak of the epidemic: a rational use of the resources requires, among other factors, predictive models able to stratify the prognosis of patients. On one hand, data of our series confirmed the shared knowledge that males are more severely affected by COVID-19 and age predicts the outcome. On the other hand, the *Brixia* score provided additional insights: the significantly different values in H- and L-scores between discharged and deceased patients suggest that if the score exceeds the value of 9 or does not drop below 7, a worse outcome can be expected.

The course of COVID-19 is characterized by sudden changes of the clinical conditions, often presenting dramatic worsening that may precipitate to intubation and transfer from COVID-19 wards to intensive care units. The prediction of these events is essential in order to achieve timely intensification of care.

The results of this study show that the observation of the oscillations of *Brixia* score on serial CXR obtained during the hospital stay may contribute to the prediction, in particular when the score increases by 3 or more points.

This study has limitations. First of all, we did not account for comorbidities that may have influenced the course of the disease and, in some cases, the imaging findings. According to the Italian National Institute of Health (ISS), 48.6% of the COVID-19 patients who died in Italy until March 20 had 3 or more comorbidities. However, it is not clear which comorbidities may impact on the outcome and which relative weight should be assigned to the different disease entities [12, 13]. Moreover, the causes of death in the 276 patients died during hospitalization were not recorded, because, at the peak of the epidemic, the local institutional guidelines and the logistics did not permit diagnostic autopsies. Vascular complications due to COVID-19-related coagulopathy, rather than respiratory distress, may account for some of the deaths. Many clinical and laboratory data are missing in a significant number of patients, and thus, they could not be implemented in the statistical analysis. Finally, we built predictive models based solely on demographic and radiological data: additional studies on larger series should be encouraged to try to build more refined models including clinical and laboratory findings.

## Conclusion

The *Brixia* score can be easily implemented in the routine reporting of CXR exams in COVID-19 patients. As this score correlates strongly with disease severity and outcome, it may support the clinical decision-making, particularly in the peak of the epidemic, when patients with moderate-to-severe signs and symptoms prevail. The results of this study suggest that the *Brixia* score should be incorporated in a prognostic model, which is essential in resource-constraint scenarios.

## Abbreviations

AUC: Area under the curve
COVID-19: Coronavirus disease 2019
CXR: Chest X-ray
ED: Emergency department
HR: Hazard ratio
ICU: Intensive care unit
IQR: Interquartile range
IRB: Institutional review board
OR: Odds ratio
ROC: Receiver operating characteristic
RT-PCR: Reverse transcriptase polymerase chain reaction
